# Integra® Dermal Regeneration Matrix: A Versatile Solution for Complex Soft Tissue Sarcoma Reconstruction in the Hand

**DOI:** 10.7759/cureus.40553

**Published:** 2023-06-17

**Authors:** Ramy Samargandi

**Affiliations:** 1 Orthopedic Surgery Department, Faculty of Medicine, University of Jeddah, Jeddah, SAU; 2 Orthopedic Surgery Department, Centre Hospitalier Régional Universitaire (CHRU) de Tours, Tours, FRA

**Keywords:** case report, reconstruction, integra, wide excision, neoadjuvant radiotherapy, hand tumor, soft tissue sarcoma, undifferentiated pleomorphic sarcoma

## Abstract

This case report describes the successful use of Integra® (Integra LifeSciences Corporation, Plainsboro, New Jersey, United States), an artificial skin substitute, for the reconstruction of soft tissue defects following soft tissue sarcoma resection. We present a case of a 75-year-old female presented with a progressively enlarging lesion on her right hand. Imaging revealed tumor involvement in the extensor tendons and adjacent to the index finger tendon. A percutaneous biopsy confirmed an undifferentiated pleomorphic sarcoma. The patient underwent neoadjuvant radiotherapy followed by wide excision of the tumor. Integra® dermal regeneration matrix was utilized to cover the exposed bone during the surgical procedure. This allowed for wound closure and provided a favorable environment for tissue regeneration and subsequent split-thickness skin graft. Complete wound healing was obtained. Regular follow-up examinations showed no evidence of local recurrence or secondary lesions after one year. The successful use of Integra®, in this case, demonstrates its efficacy as a reconstructive option for complex hand sarcomas. It offers immediate wound coverage and promotes tissue regeneration, thereby avoiding the need for more extensive treatment modalities with associated donor-site morbidity. The utilization of Integra® resulted in high patient satisfaction and excellent recovery. This case highlights the importance of utilizing innovative techniques and materials in achieving optimal outcomes in challenging hand sarcoma reconstructions.

## Introduction

Soft tissue sarcomas are rare heterogeneous groups of solid malignancies arising in connective tissues and derived from mesenchymal cell origin that can occur in various locations throughout the body, including the extremities [1]. Soft tissue sarcomas are often treated with a multidisciplinary approach, including limb salvage surgery with a wide excision in order to obtain negative margins combined with radiotherapy to improve local control and currently considered the gold standard procedure for the treatment of soft tissue sarcomas that arise in the extremities [2-5]. Among the challenging cases are those involving the dorsal aspect of the hand, where the intricate anatomy and functional demands pose unique difficulties in both tumor resection and subsequent reconstruction [6]. In such cases, the use of innovative techniques and materials becomes crucial to achieve optimal outcomes.

This case report presents a patient with soft tissue sarcoma located in the dorsal aspect of the hand, involving the overlying skin and resulting in exposure to the underlying bone. We describe the successful application of an artificial skin substitute, Integra® (Integra LifeSciences Corporation, Plainsboro, New Jersey, United States), for coverage of the exposed bone following tumor resection. The use of Integra®, in this case, facilitated wound closure, promoted tissue regeneration, and ultimately restored functionality to the hand.

## Case presentation

**Figure 1:**
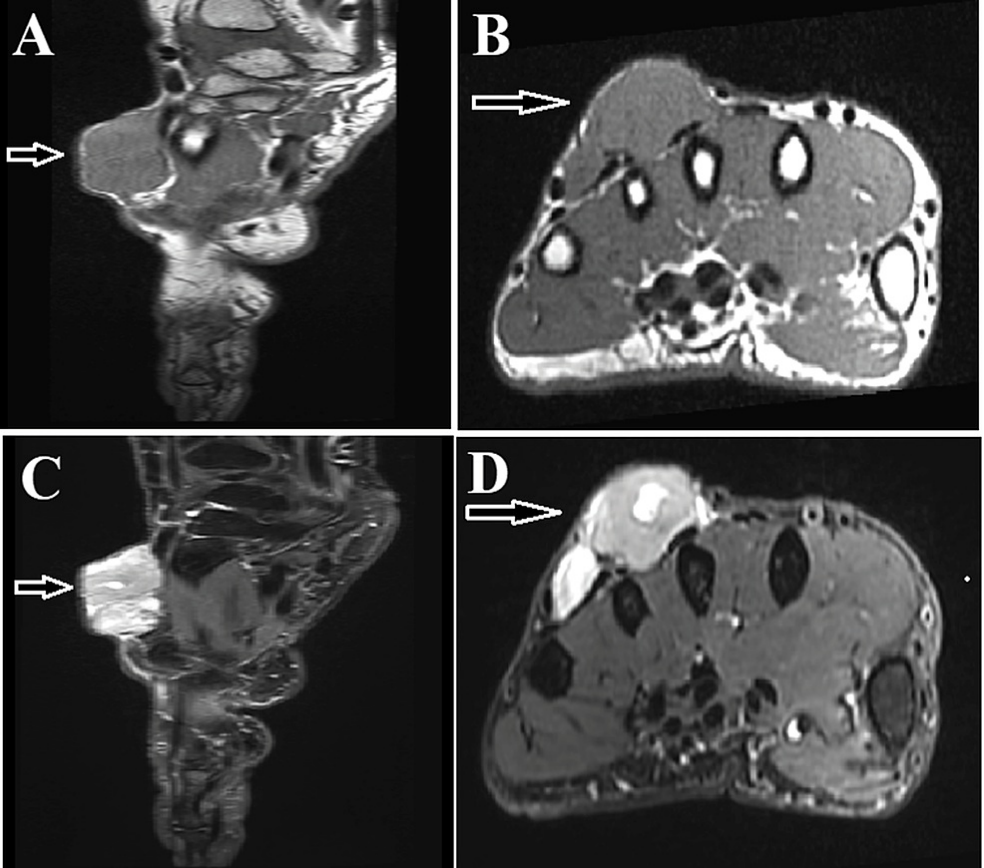
The magnetic resonance imaging (MRI) of the right hand demonstrates a lesion with low signal intensity in the T1-weighted image and a heterogeneous, high signal intensity in the T2-weighted image on the dorsum of the hand (indicated by white arrows). (A) Sagittal T1-weighted MRI image, (B) Axial T1-weighted MRI image, (C) Sagittal fat-suppressed T2-weighted image, (D) Axial fat-suppressed T2-weighted image.

A 75-year-old right-handed female patient with a history of hypertension, dyslipidemia, and hypothyroidism was initially referred to our institution in February 2022 due to a gradually enlarging lesion on the dorsal aspect of her right hand, measuring 5.4 cm in the largest dimension. Magnetic resonance imaging (MRI) revealed a heterogeneous lesion invading the extensor tendons of the third, fourth, and fifth digits and close to the extensor tendon of the index finger (Figure 1).

A percutaneous biopsy confirmed the presence of an undifferentiated malignant tumor composed of spindle and epithelioid cells, consistent with a grade 2 undifferentiated pleomorphic sarcoma according to the Fédération Nationale des Centres de Lutte Contre Le Cancer (FNCLCC) grading system [7]. After discussing the diagnosis with the patient and providing psychological support, a staging workup revealed no evidence of metastasis. The case was presented at the orthopedic oncology tumor board, and a multidisciplinary decision was made to proceed with neoadjuvant radiotherapy followed by wide excision and reconstruction using Integra® artificial dermis without reconstruction of extensors tendons. Tendon reconstruction was not planned due to considerations such as the patient's advanced age, comorbidities, low functional demand, and the risk of recurrence. This decision was thoroughly discussed with the patient prior to the operation.

**Figure 2:**
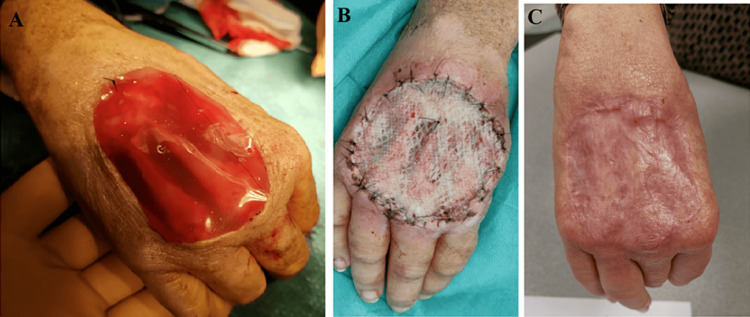
Local image of the right hand (A) After sarcoma resection and application of Integra® for covering exposed metacarpal bones which were fixed with sutures, (B) Split-thickness skin graft three weeks after the initial resection obtained from the right thigh, (C) Complete wound healing.

The patient underwent neoadjuvant radiotherapy with a total dose of 50 Gy in two fractions, completed in May 2022. The resection of the tumors involved sacrificing extensor tendons, periosteum, and a layer of dorsal interossei muscles to achieve negative margins. We attempted to preserve the second extensor tendon, but intraoperative observations indicated that conserving it would be challenging due to the tumor's close proximity. Considering our concern about not achieving negative margins, we made the decision to include it in the resection. Following the tumor resection, the metacarpal bones (second-fifth) were exposed, and Integra® was used to cover the soft tissue defect (Figure 2). Complete resection with negative margins was obtained (R0) with an estimated 10% viable residual tumor. Postoperative immobilization was provided with a splint. Three weeks later, the patient underwent reoperation for a split-thickness skin graft (STSG) taken from the right thigh in outpatient surgery. Rehabilitation commenced five days after the skin graft. Complete wound healing was obtained (Figure 2).

**Figure 3:**
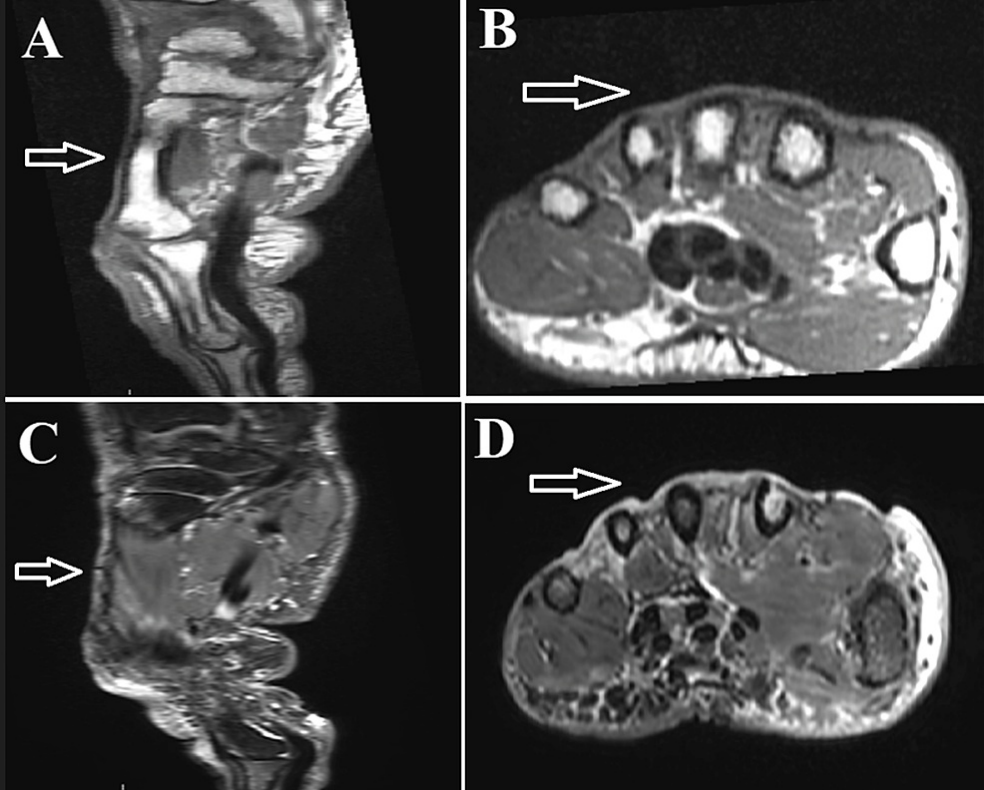
Magnetic resonance imaging (MRI) of the right hand at one-year follow-up showing no recurrence at the site of initial lesion (white arrows). (A) Sagittal T1-weighted MRI image, (B) Axial T1-weighted MRI image, (C) Sagittal fat-suppressed T2-weighted image, (D) Axial fat-suppressed T2-weighted image.

After careful deliberation in the multidisciplinary tumor board, it was concluded that no additional adjuvant therapy was required. Adjuvant chemotherapy was not recommended due to the absence of distant metastasis and the patient's age. Regular follow-up with local MRI and thoraco-abdominopelvic computed tomography (CT) scans every four months revealed no evidence of local recurrence or secondary lesions after one year of follow-up (Figure 3). The patient will continue to be closely monitored for 10 years.

## Discussion

The management of soft tissue sarcomas in hand presents several challenges, including achieving complete tumor resection with negative surgical margins while obtaining wound coverage and ensuring the deliverance of radiotherapy.

Preoperative and postoperative radiotherapy is a commonly proposed treatment in the management of sarcoma along with surgery, each offering distinct benefits and risks [3,8,9]. Preoperative radiotherapy has been shown to increase the incidence of wound complications; however, it delivers a lower radiation dose to the patient, thereby reducing the risk of fibrosis, lymphedema, and joint stiffness. On the other hand, postoperative radiotherapy has demonstrated a lower risk of wound complications despite the higher radiation dose administered [10-12]. In the current case, we opted for preoperative radiation for several reasons. Firstly, we aimed to reduce the size of the lesion in order to preserve the extensor tendons that were in contact with the tumor. Secondly, given that a soft tissue reconstruction was already planned, we wanted to avoid irradiating the reconstructed tissues. Lastly, we sought to minimize the radiation dose delivered to the hand, as postoperative radiotherapy typically involves higher doses, which are associated with increased fibrosis and joint stiffness. Such outcomes could yield poorer results compared to other anatomical locations. Another noteworthy point to mention in the present case is that the risk of wound complications was anticipated to be high due to the exposure of multiple metacarpal bones. As a result, it was crucial to ensure that the patient received radiotherapy preoperatively. This decision was made because if wound complications were to occur, the patient might not be able to undergo postoperative radiotherapy within the recommended timeframe due to the presence of these complications, as observed in a previous study [13].

In cases where tumor involvement necessitates the resection of a large amount of soft tissue, the resulting bone exposure poses additional difficulties in achieving successful wound healing. After removing the periosteum, exposed bone cannot form a layer of healthy granulation tissue where skin graft can be applied directly and integrated. Additionally, it is important to consider early coverage of exposed bone to avoid bone infection and accelerate wound healing, especially in the case of sarcoma patients when adjuvant therapy is needed. Traditional reconstruction methods, such as skin grafts or local flaps, may be limited in their ability to provide durable coverage.

Integra®, a bi-layered dermal regeneration matrix, has demonstrated considerable success in various reconstructive applications, including complex wounds and soft tissue defects, as well as in cancer resection [14-16]. It acts as a scaffold for cellular infiltration, angiogenesis, and the formation of new tissue, ultimately integrating with the surrounding tissue and promoting wound healing. Its unique composition provides a stable environment that encourages tissue regeneration and minimizes scar formation [16]. Integra® is a safe and effective method to treat complex hand wounds from nonburn trauma and cancer resection with exposed bone, joints, or tendons [17]. Its use in complex hand wounds offers a reconstructive alternative to more extensive treatment options utilizing flap transfer and their accompanying donor-site morbidity.

In the present case, the use of Integra® proved to be a valuable adjunct in achieving successful wound closure and functional restoration, even with the history of neo-adjuvant radiotherapy. By covering the exposed bone, Integra® provided a protective barrier, facilitated the ingrowth of blood vessels and fibroblasts, and promoted the formation of a neodermis. This allowed for subsequent split-thickness skin grafting, resulting in a durable and aesthetically satisfactory outcome.

The successful use of Integra®, in this case, highlights its potential as an effective option in reconstructing complex hand wounds following sarcoma resection. It offers several advantages, including covering exposed bone, promoting tissue regeneration, minimizing scarring, and improving patient outcomes. Moreover, patients can benefit from definitive wound coverage within a short timeframe of 2-3 weeks following Integra® placement, as demonstrated in the presented case. Further studies and long-term follow-up are warranted to assess the durability and functional outcomes of Integra® in similar cases.

## Conclusions

The case presented demonstrates the successful utilization of Integra® as an artificial skin substitute in the reconstruction of a soft tissue sarcoma involving the dorsal aspect of the hand. The application of Integra® facilitated wound healing and promoted tissue regeneration without requiring free tissue transfer or distant flaps, which would have added additional morbidity to the patient. The case highlights the importance of innovative techniques and materials in achieving optimal outcomes in challenging reconstructive procedures.
